# Silicon Regulates Source to Sink Metabolic Homeostasis and Promotes Growth of Rice Plants under Sulfur Deficiency

**DOI:** 10.3390/ijms21103677

**Published:** 2020-05-23

**Authors:** Elise Réthoré, Nusrat Ali, Jean-Claude Yvin, Seyed Abdollah Hosseini

**Affiliations:** Centre Mondial de l’Innovation Roullier, Laboratoire de Nutrition Végétale, Pôle Stress Abiotiques, 18 avenue Franklin Roosevelt, 35400 Saint-Malo, France; Elise.Rethore@roullier.com (E.R.); Nusrat.Ali@roullier.com (N.A.); JeanClaude.Yvin@roullier.com (J.-C.Y.)

**Keywords:** transcriptional regulation, primary metabolism, hormones, nutritional stress

## Abstract

Being an essential macroelement, sulfur (S) is pivotal for plant growth and development, and acute deficiency in this element leads to yield penalty. Since the last decade, strong evidence has reported the regulatory function of silicon (Si) in mitigating plant nutrient deficiency due to its significant diverse benefits on plant growth. However, the role of Si application in alleviating the negative impact of S deficiency is still obscure. In the present study, an attempt was undertaken to decipher the role of Si application on the metabolism of rice plants under S deficiency. The results showed a distinct transcriptomic and metabolic regulation in rice plants treated with Si under both short and long-term S deficiencies. The expression of Si transporters *OsLsi1* and *OsLsi2* was reduced under long-term deficiency, and the decrease was more pronounced when Si was provided. The expression of *OsLsi6*, which is involved in xylem loading of Si to shoots, was decreased under short-term S stress and remained unchanged in response to long-term stress. Moreover, the expression of S transporters *OsSULTR* tended to decrease by Si supply under short-term S deficiency but not under prolonged S stress. Si supply also reduced the level of almost all the metabolites in shoots of S-deficient plants, while it increased their level in the roots. The levels of stress-responsive hormones ABA, SA, and JA-lle were also decreased in shoots by Si application. Overall, our finding reveals the regulatory role of Si in modulating the metabolic homeostasis under S-deficient condition.

## 1. Introduction

Nutritional disorder is one of the major constraints for plant growth and development. Sulfur (S) is considered as an indispensable macronutrient for crop plants, since it is a constituent of the amino acids cysteine and methionine, and hence of proteins, but also of secondary metabolites such as vitamins or defense compounds such as glucosinolates [1,2]. The S requirement is usually between 0.1% and 0.5% of plant dry weight to ensure optimal plant growth [1]. Upon S deficiency, shoot growth is inhibited, leading to a decrease in the shoot/root ratio. The lack of S can also inhibit the uptake of other nutrients, such as carbon and nitrogen, resulting in deficiencies and a decrease in protein synthesis. Moreover, S deprivation decreases the level of S-containing defense compounds, such as glutathione, hydrogen sulfide (H_2_S), or many secondary metabolites [3]. The regulation of S assimilation is pivotal under control condition but also under abiotic stresses, such as drought stress or heavy metal toxicity [4,5]. Most of the abiotic stresses increase the level of reactive oxygen species, which can be deleterious at high concentration. One of the enzymatic defense systems is the ascorbate–glutathione cycle, which derives from S metabolism pathways [6]. The balance between reduced glutathione and oxidized glutathione is critical under drought stress and has been well reported in previous studies [4,7].

The response of plants to S deficiency as well as improving S uptake and utilization has been studied for decades [8,9]. The early response to S limitation includes the overexpression of the genes involved in sulfate transport and assimilation [10] with a tandem increase in the catabolism of S-containing compounds and a decrease in the synthesis of secondary metabolites [2]. The efforts of Smith et al. resulted in pioneering work to study the regulation of S transporters in the plant model *Arabidopsis thaliana* [11,12,13]. In this species, four main groups of sulfate transporters were identified: the *AtSULTR1* family corresponds to high-affinity transporters and the *AtSULTR2* family corresponds to low-affinity transporters, whereas *AtSULTR4* would encode vacuolar transporters and *AtSULTR3* would encode plastidial transporters and/or co-activators of other transporters [14]. In rice, 14 candidate *OsSULTR* genes were identified and classified according to their homology with *Arabidopsis* transporters [15,16]. However, to our knowledge, their precise function and their transcriptional response under short or long-term S deficiency have not yet been evaluated. Under long-term S deficiency, the capacity of plants to regulate S homeostasis through the increase of lateral root growth and induction of S transport/assimilation is overwhelmed. Then, the photosynthetic activity of plants declines due to a decrease in chlorophyll content, reduction in protein abundance, and lower enzymatic activity of photosystem I as well as the Rubisco protein [17]. This leads to the shortening of the life cycle and induces an early senescence [10,18].

Plants have developed a wide range of mechanisms to maintain their productivity and thus ensure their survival under nutritional disorder. Among the mineral nutrients, silicon (Si) is considered as a non-essential element for crop plants [19]. However, several studies highlighted that Si exerts a beneficial effect under different environmental stresses in many plant species. In this case, the positive effect of Si in alleviating mineral nutrient deficiency in different plant species has been extensively reported. For instance, Si has been shown to mitigate the potassium (K) deficiency in soybean [20] and sorghum plants grown under low K condition [21,22]. Moreover, we have shown in our previous study that Si delayed leaf senescence and maintained the growth of barley plants exposed to combined S and osmotic stress [23]. The regulatory role of Si in alleviating mineral nutrition has been also reported under nitrogen (N) [24], phosphorus (P) [25,26], and magnesium (Mg) [27] deficiencies. However, the relevance of Si solely under S stress has not yet been well investigated. To benefit from Si, plants use two different Si transporters which have been identified in many crops such as rice [19], wheat [28], barley [29], and maize [30]. These transporters are known to play a specific role in the uptake and translocation of Si from roots to shoots. In rice, two distinct transporters—*OsLsi1* and *OsLsi2*—have been implicated in the uptake and transport of Si from roots cells to the apoplast [31], while a third transporter *OsLsi6* is responsible for unloading and distribution of Si from the xylem to leaf tissues [32]. The expression of Si transporters, specifically *Lsi1* and *Lsi2*, is shown to be regulated under stress conditions in different crops. For example, the short-term application of drought or ABA decreased the expression of both genes under a Si-free condition [33,34]. It is worthy to highlight that Si also modifies the expression of the genes involved in the uptake/translocation of mineral nutrients such as N [24], P [35], and K [21]. However, these research studies have investigated the role of Si in a long-term condition either at a certain time point or under different doses of Si. Only a recent effort by Jang et al. in 2018 reported the effect of short-term Si application in rice plants [36]. Even though Si is considered to have a limited direct role under non-stress condition [37], this study has focused on the effects of Si on mineral nutrition under control conditions, but not under mineral deficiency [36].

In this study, an effort was undertaken to investigate whether and how the application of Si attenuates both short-term and long-term S deficiency. In this regard, hydroponically grown rice plants were exposed to short (6 h, 12 h, 24 h) as well as long-term (3 days and 15 days) S deficiency, while Si was provided at the same time of S deficiency. The distinct regulatory role of Si under short-term and long-term application in S-deficient plants was considered using physiological, biochemical, and gene expression analyses.

## 2. Results

### 2.1. Silicon Transcriptionally Changed the Expression of S and Si Transporters under Sulfur Deficiency

**Figure 1:**
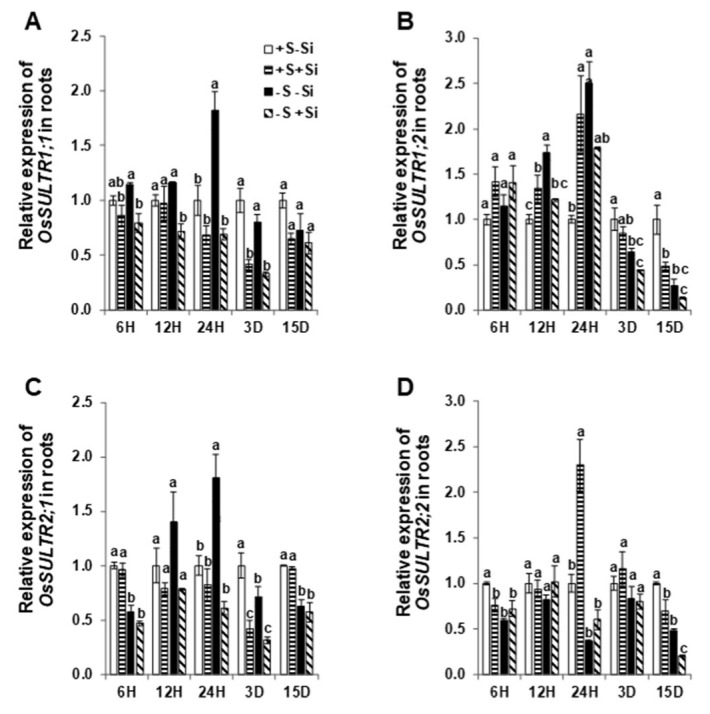
Influence of silicon application on the expression levels of high and low-affinity sulfur transporters in rice roots exposed to S deficiency. (**A**) Relative *OsSULTR1;1* mRNA level in roots; (**B**) relative *OsSULTR1;2* mRNA level in roots; (**C**) relative *OsSULTR2;1* mRNA level in roots; and (**D**) relative *OsSULTR2;2* mRNA level in roots. Plants were grown in hydroponic culture under either low (0 mM) or normal S (1.5 mM) as well as 2 mM of Si supply. Roots were harvested for the gene expression analysis after 6 h, 12 h, 24 h, 3 days, or 15 days of the treatments. Relative gene expression at each time point was normalized to the expression of +S/–Si plants. Bars indicate mean ± SEM. Different letters denote significant difference according to ANOVA followed by the Student–Newman–Keuls (SNK) test (*p* < 0.05; *n* = 4). Statistical analysis was performed independently for each time point.

In order to better understand whether Si could transcriptionally regulate S transporters, the expression pattern of selected *OsSULTR* family genes, *OsSULTR1;1, OsSULTR1;2, OsSULTR2;1,* and *OsSULTR2;2* were monitored in rice roots following short (6 h, 12 h, 24 h) and long-term (3 days and 15 days) Si application and S deficiency (Figure 1). Under ample S supply, the expression of high-affinity SO_4_^2^^−^ transporters *OsSULTR1;1* and *OsSULTR1;2* was not significantly impacted by Si except for a decrease in the expression of *OsSULTR1;1* after 3 days of Si supply and a transient increase in the expression of *OsSULTR1;2* after 24 h, which was followed by a decrease at 15 days (Figure 1A,B). The expression of the low-affinity transporters *OsSULTR2;1* and *OsSULTR2;2* followed the same pattern as that of *OsSULTR1;1* and *OsSULTR1;2* (Figure 1C,D). Under S deprivation alone, the expression of both *OsSULTR1;1* and *OsSULTR2;1* was sharply induced already after 24 h of S deficiency compared to the ample S supply, together with *OsSULTR1;2* after 12 and 24 h, whereas the expression of *OsSULTR1;2* and *OsSULTR2;1* was down-regulated over long-term S deprivation (3 days and 15 days) (Figure 1A–C). The expression of *OsSULTR2;2* was not affected by low S compared to ample S supply, except a slight decrease at 6 h and 15 days of S deprivation (Figure 1D). The expression of *OsSULTR1;1* gene tended to decrease by Si application already after 6h and persisted until 3 days under the lack of S compared to S-deficient plants that did not receive Si (Figure 1A). An early decrease in the expression level of *OsSULTR1;2* was also observed under S deficiency, but only after 12 h of Si supply (Figure 1B). The expression level of *OsSULTR2;1* was significantly decreased after 24 h to 3 days of S deficiency in response to Si application and that of *OsSULTR2;2* after 15 days (Figure 1C,D). Overall, these results showed that Si differentially changes the expression of high and low-affinity S transporters in response to short and long-term S deficiency.

**Figure 2:**
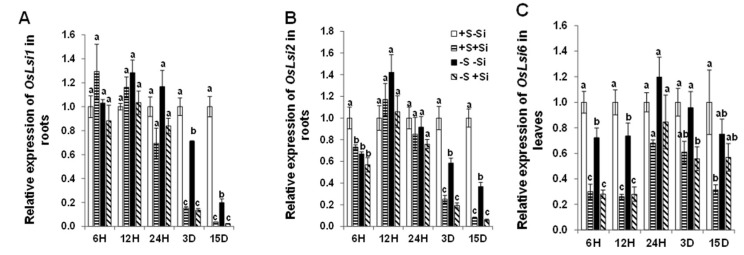
Influence of silicon application on the expression levels of silicon transporters in rice roots and shoots exposed to sulfur deficiency. (**A**) Relative *OsLsi1* mRNA level in roots; (**B**) relative *OsLsi2* mRNA level in roots; and (**C**) relative *OsLsi6* mRNA level in shoots. Plants were grown in hydroponic culture under either low (0 mM) or normal S (1.5 mM) as well as 2 mM of Si supply. Roots were harvested for the gene expression analysis after 6 h, 12 h, 24 h, 3 days, or 15 days of treatments. Relative gene expression at each time point was normalized to the expression of +S/–Si plants. Bars indicate mean ± SEM. Different letters denote significant difference according to ANOVA followed by the SNK test (*p* < 0.05; *n* = 4). Statistical analysis was performed independently for each time point.

We further examined the expression of Si transporters *OsLsi1* and *OsLsi2*, which cooperatively mediate the uptake and the accumulation of Si in rice plants [31]. Under ample S supply, the expression level of *OsLsi1* did not change under short-term conditions, but it declined at 3 and 15 d by Si application (Figure 2A). The expression patterns of *OsLsi2* was already decreased after 6h, remained unchanged after 12 h and 24 h, and was suppressed under long-term conditions (3 days and 15 days) by Si supply (Figure 2B). In addition, the expression level of *OsLsi6* was decreased both under short and long-term Si supply (Figure 2C). Under S deficiency, the expression of both *OsLsi1* and *OsLsi2* genes were significantly decreased after 3 and 15 days of S stress (Figure 2A,B). The effect of Si on the expression of both genes was identical irrespective of either treatments, with a sharp decline after 3 days of Si application. Conversely, the expression level of *OsLsi6*, which mediates the transport of Si from xylem to leaves [32], was significantly decreased only under short-term (6 h and 12 h) S stress, irrespective of the Si supply. The expression of this gene in both Si-treated and non-Si-treated plants remained in general high when S deficiency prolonged (24 h onwards). These data show that Si transporters were differentially expressed in roots and shoots under low S supply and Si treatment.

### 2.2. Si Changed the Nutritional Homeostasis in Root and Shoot under Control and S Deficiency

Elemental analysis was performed after 15 days of Si application in both roots and shoots of ample S and in S-deprived plant using inductively coupled plasma mass spectrometry. The macro-elements are presented in Table 1. In roots and under ample S supply, Si application provoked a significant increase in the concentration of Si, total Mg, and SO_4_^2^^−^ ions. At the same time, P concentration was significantly reduced in the roots by Si application. Under S deficiency, only the concentration of Si was significantly increased by Si supply. In shoots and under low S supply, the level of Si significantly increased when Si was supplemented, whereas the level of all other elements except K and NO_3_^−^ were significantly decreased. Two-way ANOVA revealed that there was a significant interaction between S and Si in the shoot Si and NO_3_^−^ values (*F*-value = 17.7, Pr(>F) = 0.00122 for Si and *F*-value = 9.156, Pr(>F) = 0.012767 for NO_3_^−^) (Supplementary Table S1).

### 2.3. Application of Si Differentially Regulated Primary Metabolism in Root and Shoot under S Deficiency

**Figure 3:**
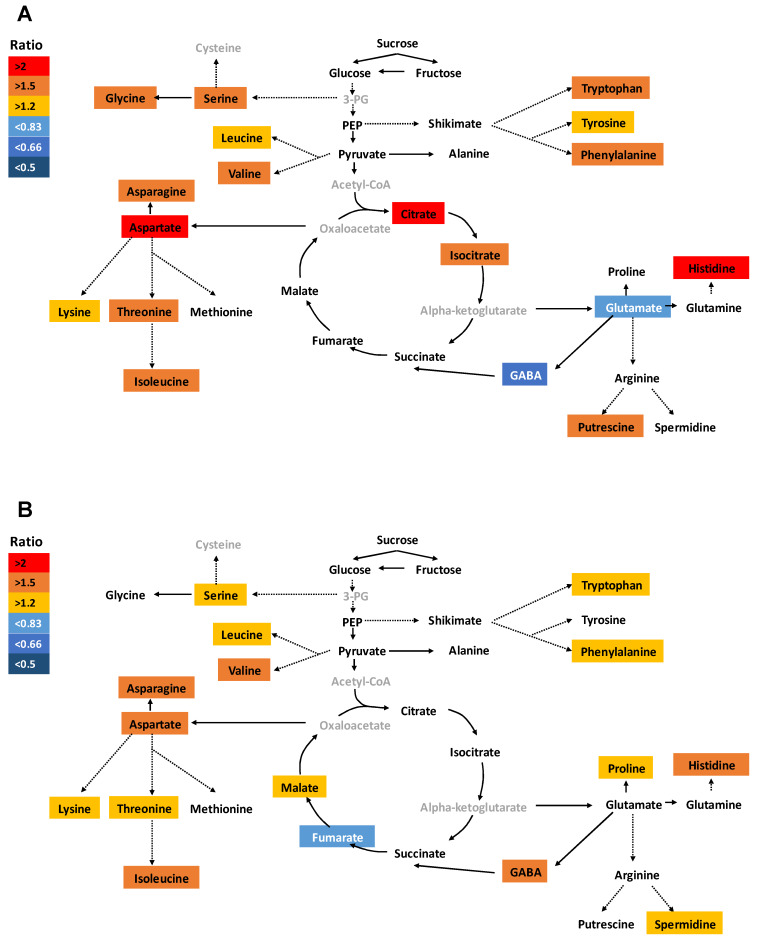
Influence of sulfur deficiency on the concentration of the primary metabolites in rice roots and shoots. Schematic representation of primary metabolites in response to S deficiency (**A**) for root, and (**B**) for shoot. Plants were grown in hydroponic culture under either low (0 mM) or normal S (1.5 mM). Roots and shoots were harvested for metabolite profiling after 15 days of treatments. Metabolites were colored according to the ratio of S deficient to S sufficient plants (light blue to dark blue: decrease; yellow to red: increase). The gray color indicates the unquantified metabolites. Solid arrows denote single reactions, whereas dotted arrows indicate that some intermediate metabolites are not shown. Only the metabolites that presented statistically significant differences are presented. Statistical analysis was done using ANOVA followed by the SNK test (*p* < 0.05; *n* = 4).

Due to the undeniable role of metabolites in plant growth and development, the effect of Si was further investigated on primary metabolites such as amino and organic acids, soluble sugars, as well as polyamines. In this study, we focused mainly on the last time point (15 days), as the metabolites are generally more affected under long-term stress application. Under ample S supply and in roots, a significant increase in glutamine level was observed, while levels of leucine, lysine, gamma-aminobutyric acid (GABA), arginine, fumarate, and putrescine were significantly decreased (Supplementary Figure S1A). In shoots of S sufficient plants, the concentration of methionine, fumarate, and shikimate were significantly increased, whereas the level of amino acids histidine, serine, lysine, threonine, tyrosine, phenylalanine, tryptophan, and asparagine were decreased together with putrescine after Si application (Supplementary Figure S1B). However, the total amount of amino acids did not change in spite of Si supply (Supplementary Figure S2). Under S deficiency alone, the root concentration of glutamate and GABA were significantly decreased compared to ample S condition, while the majority of amino acids, together with the organic acids citrate and isocitrate, were significantly accumulated in response to S deficiency (Figure 3A). This increase also held true when we recorded the total amino acid accumulation in roots (Supplementary Figure S2A). In S-deficient plants, almost all amino acids significantly accumulated in shoots compared to ample S condition and with a tandem increase in the level of malate and spermidine (Figure 3B). The level of soluble sugars glucose, fructose, and sucrose were not significantly impacted by the stress in both roots and shoots.

**Figure 4:**
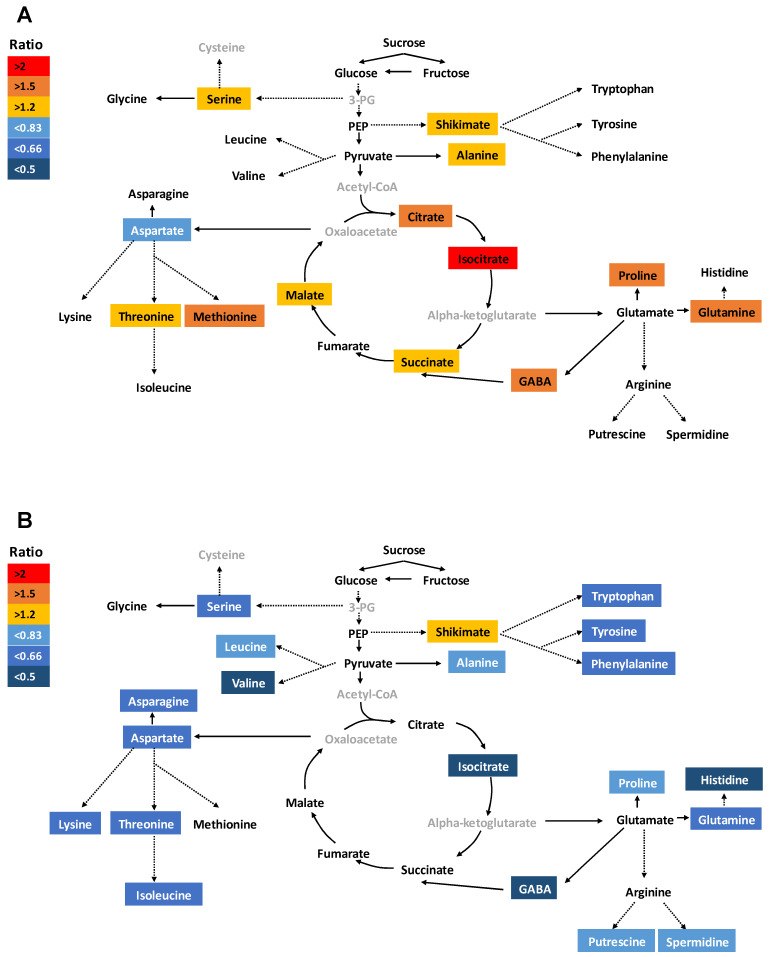
Influence of silicon application on the concentrations of primary metabolites in rice roots and shoots exposed to sulfur deficiency. Schematic representation of primary metabolites under S deficiency and in response to Si supply (**A**) for root and (**B**) for shoot. Plants were grown in hydroponic culture under low S (0 mM) as well as 2 mM of Si supply. Roots and shoots were harvested for metabolite profiling after 15 days of treatments. Metabolites were colored according to the ratio of S-deficient treated with Si to sole S-deficient plant (light blue to dark blue: decrease; yellow to red: increase). The gray color indicated the unquantified metabolites. Solid arrows denote single reactions, whereas dotted arrows indicate that some intermediate metabolites are not shown. Only the metabolites that presented statistically significant differences were presented. Statistical analysis was done using ANOVA followed by the SNK test (*p* < 0.05; *n* = 4).

In the roots of S-deprived plants, Si increased the level of most of the amino acids (threonine, methionine, serine, proline, alanine, glutamine, and GABA) and organic acids (shikimate, citrate, isocitrate, succinate, and malate) without any changes in polyamine levels. Aspartate was the only amino acid whose level was decreased by Si application compared to S-deficient plants (Figure 4A). We observed a significant interaction between S and Si for amino acid GABA and proline in roots (*F*-value = 24.79, Pr(>F) = 0.0004 and *F*-value = 10.42, Pr(>F) = 0.008, respectively) as well as for organic acids citrate and isocitrate (*F*-value = 11.23, Pr(>F) = 0.006 and *F*-value = 21.37, Pr(>F) = 0.0006, respectively) (Supplementary Table S1). The total amino acid amount was significantly increased in roots but remained unchanged in shoots (Supplementary Figure S2). Our ANOVA analysis showed a significant interaction between S and Si for total amino acids in shoots (*F*-value = 7.91, Pr(>F) = 0.0169) (Supplementary Table S1). Similarly, the concentration of soluble sugars was not significantly affected by either treatments in both shoots and roots. In shoots, the S-deprived plants showed a significant reduction in the levels of most of the amino acids together with isocitrate, putrescine, and spermidine when Si was provided (Figure 4B). Shikimate was the only metabolite that significantly accumulated in shoots under Si treatment. Altogether, these results clearly demonstrate that Si mediates distinct metabolic homeostasis in roots and shoots under the lack of S.

### 2.4. Application of Si Modulated Hormonal Responses in Shoots under S Deficiency

**Figure 5:**
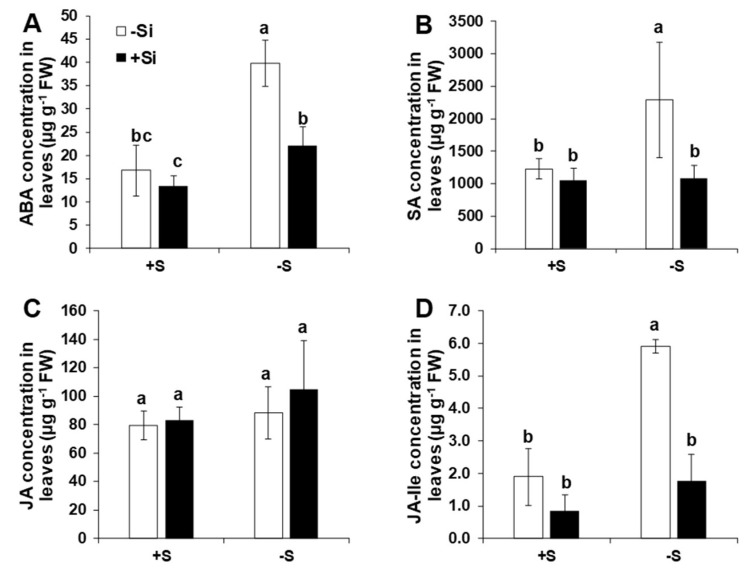
Influence of silicon application on the concentration of phytohormones in rice shoots exposed to sulfur deficiency. (**A**) Abscisic acid (ABA) concentration; (**B**) salicylic acid (SA) concentration; (**C**) jasmonic acid (JA) concentration; and (**D**) jasmonoyl-isoleucine (JA-Ile) concentration. Plants were grown in hydroponic culture under either low (0 mM) or normal S (1.5 mM) as well as 2 mM of Si supply. Shoots were harvested for hormonal analysis after 15 days of treatments. Bars indicate mean ± SD. Different letters denote significant difference according to ANOVA followed by SNK test (*p* < 0.05; *n* = 4).

Due to important function of phytohormones under nutrient deficiency [38], hormonal profiling was performed only for the last time point. Under ample S supply, the application of Si had no significant effect on the level of the measured hormones abscisic acid (ABA), salicylic acid (SA), jasmonic acid (JA), and its derivate jasmonoyl-isoleucine (JA-Ile) (Figure 5). Except JA, the levels of ABA, SA, and JA-Ile were all up-regulated in response to low S and compared to ample S supply. Interestingly, application of Si significantly reduced the levels of phytohormones ABA, SA, and JA-Ile under the lack of S (Figure 5A,B,D). For ABA and JA-Ile, we found a significant interaction in our ANOVA analysis between S and Si (*F*-value = 11.03, Pr(>F) = 0.006 and *F*-value = 18.66, Pr(>F) = 0.0019, respectively) (Supplementary Table S1). These data show the role of Si in regulating the phytohormone levels under the lack of S in rice plants.

### 2.5. Application of Si Improved Plant Growth and Development under Control Condition and S Deficiency

**Figure 6:**
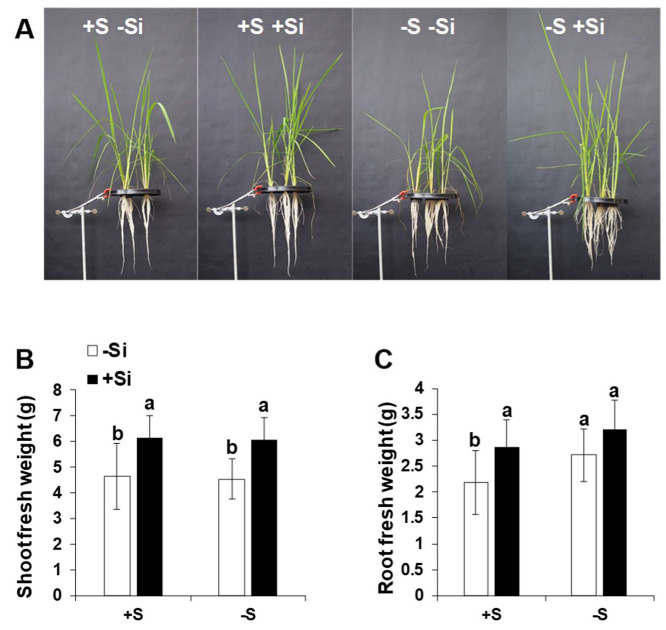
Influence of silicon application on shoot and root biomass of rice plants exposed to sulfur deficiency. (**A**) Monitoring shoot phenotype under ample and deficient S supply and Si application, (**B**) shoot fresh weight, and (**C**) root fresh weight. Plants were grown in hydroponic culture under either low (0 mM) or normal S (1.5 mM) as well as 2 mM of Si supply. Roots and shoots were harvested after 15 days of treatments. Bars indicate mean ± SD. Different letters denote significant difference according to ANOVA followed by the SNK test (*p* < 0.05; *n* = 4).

The effect of sulfur deficiency and silicon application on growth and its related parameters were further determined after 15 days of S stress and in response to Si supplement. Shoot fresh weight did not differ under ample S and S deficient condition. This could be due to relatively long period of growing plants under ample S before subjecting them to S stress, which could be sufficient for S remobilization in the plant. However, Si application increased the shoot fresh weight in spite of S nutrition (Figure 6A,B). Two-way ANOVA showed the significant difference for Si treatment in the shoot biomass (*F*-value = 29.622, Pr(>F) = 2.21 × 10^−6^) (Supplementary Table S1). This effect was also held true for root fresh weight (Figure 6C). As expected, S-deficient plants showed significantly higher root fresh weight in comparison to their corresponding S-supplied plants, which is likely a mechanism to increase the capacity of the plant to uptake S. A slight but not significant increase in root fresh weight was also observed in the S-deprived plant that did receive Si compared to ample S condition. A significant effect of S and Si was also found for this parameter (*F*-value = 7.517, Pr(>F) = 0.008804 for S and *F*-value = 13.581, Pr(>F) = 6.23 × 10^−4^ for Si) (Supplementary Table S1). Altogether, the results show that Si positively influenced the growth of rice plants under both sufficient and insufficient S supply, and root growth was moderately responding to Si application.

## 3. Discussion

In spite of the impressive number of studies that have shown the role of Si in alleviating nutritional stresses, the exact role of Si and its relevance under S deficiency still remains unclear. In this work, an attempt was undertaken to explore the response of hydroponically grown rice plants to short and long-term application of Si under either ample S or S-deficient conditions. The aim was to decipher whether and how Si could eventually change the plant metabolism in order to cope with long-term nutritional deficiencies. We observed that the expression of Si transporters was decreased in roots when S was depleted for long-term, and the decline was even more pronounced by Si application. In return, Si decreased the expression of S transporters under short-term S deficiency. We also showed that under long-term S deficiency, Si allocated the primary metabolites more to the roots, while it retained their level in an optimum range in shoots.

### 3.1. Si Supply Differentially Changes the Expression of S and Si Transporters in Response to Short and Long-Term S Deficiencies

To our knowledge, the expression profiles of sulfate transporters under control and stress conditions have been well-investigated in different crop plants, but not extensively in rice. In *Arabidopsis*, *AtSULTR1;1* and *AtSULTR1;2* were shown to have a partially non-redundant function [39] with a predominant role of *AtSULTR1;2* in sulfate uptake under control condition [40]. While the expression of *AtSULTR1;1* was dependent on sulfate concentration, the regulation of *AtSULTR1;2* was controlled by metabolic demand and photoperiod [41]. In the present work, we found that the transcript levels of *OsSULTR1;1*, *OsSULTR1;2,* and *OsSULTR2;1* were all up-regulated in response to short-term S deficiency (12 h to 24 h) but not under long-term S deprivation, whereas *OsSULTR2;2* did not respond to the lack of S. This is in agreement with previous work in *Arabidopsis* where the expression of *AtSULTR2;2* was not induced under short-term S deprivation [42]. This indicates that the induction of *OsSULTR1;2* might be responsible for sensing the S limitation in rice, which has also been identified as potential primary sensor of S deficiency in *Arabidopsis* [2,43]. Moreover, our finding is contradictory to those of Buchner et al., where they found that the expression of *TaSULTR1;1* and *TaSULTR2;1* significantly up-regulated from 5 days to a longer period of S deprivation [44]. This varying observation could be due to the experimental set-up (time of S application) and more importantly the species-dependent responses and also considering the fact that rice contains two Casparian strips which might influence on the transport of minerals compared to the other monocotyledons [45]. Indeed, compared to cereals such as barley and maize, which uptake Si by *Lsi1* and *Lsi2* at different cell layers, in rice root, Si uptake is cooperatively mediated by *Lsi1* and *Lsi2*, which are localized at the distal and proximal side, respectively, of both the exodermis and endodermis [31]. Nevertheless, further investigations need to be considered to understand concretely the crop-specific responses to S deficiency.

Si was also shown to change the expression level of the genes involved in the uptake and transport of individual nutrients and under different nutritional fluctuations such as N [46], P [35], and K [21] deficiencies. To our best knowledge, the relevance of Si nutrition under S deficiency has not been well investigated. In the pioneering work, we have shown that in barley plants subjected to concomitant S and osmotic stress, Si induced the expression of S transporter HvST1.1, resulting in a higher uptake of SO_4_^2−^, regulated ABA metabolism, and induced leaf senescence retardation [23]. Although another stress has interfered together with S stress, however, the first evidence on the changes in the expression of S transporters by Si application was encouraging to decipher further the relevance of Si nutrition in modulating the S transporter. In the present work, the expression of *OsSULTR1.1* and *OsSULTR2.1* was decreased by Si under short-term S (12 h, 24 h, and 3 days) deficiency compared to the S-deficient plant that did not receive Si, while their expression remained unchanged under long-term S deficiency.

Chen et al. have previously shown in sorghum that Si decreased the expression of *AKT1* and *HAK5* transporters under K deficiency, but that of xylem loading was improved, which was likely through the up-regulation of *SKOR* genes and the maintenance of higher water flow through aquaporins [21]. They showed that Si did not improve the K concentration in the plant, but it rather improved the plant water status. In addition, Si was shown to change the expression of P transporters in wheat [25] and rice [35] plants. Another reason for the down-regulation of S transporters could be due to the higher accumulation of Si in the shoots of S-deficient plants. This was shown by Hu et al., in a rice plant using an *Oslsi1* mutant [35]. The authors showed that the increase of shoot Si resulted in the down-regulation of the P transporter and a decrease of P uptake. This is in agreement with our finding where the application of Si suppressed the expression of S transporters, which then leads to a decrease in S uptake under long-term S deficiency. Altogether, our results provide further evidence that Si modulates the expression of both low and high-affinity S transporters depending on the duration of stress, which eventually enables plants to positively deal with different S status.

We believe that in the present work and under the lack of S, Si does not improve S status within the rice plants, but it most likely decreases the expression levels of S transporters that are associated with a global decrease in the stress levels displayed by the regulation of primary metabolites.

In essence, rice has been categorized as a Si-accumulating species, thus demanding a high amount of Si for its sustainable productivity. The pioneer work by Ma and Yamaji in 2015 demonstrated two different types of Si transporters in rice [19]. Basically, Si enters the roots via a passive channel-type transporter (*Lsi1*), which have many homologs in other crops such as barley [47], wheat [28], maize [30], and cucumber [48]. Then, an efflux transporter (*Lsi2*) mediates the loading of Si out of the plant cells and into the xylem [19]. These two transporters have shown different expression patterns depending on the type of stresses and plant species. For instance, Yamaji and Ma showed that drought stress reduced the expression of *OsLsi1* and *OsLsi2*, while Mitani et al. showed that the expression of *HvLsi2* and *ZmLsi2* was down-regulated by Si supply [30,33,34]. In another study in rice, Mitani-Ueno et al. showed that the down-regulation of rice Si transporters *OsLsi1* and *OsLsi2* has led to an accumulation of Si in shoot [49]. In the present work, the expression of both *OsLsi1* and *OsLsi2* was suppressed by Si under long-term S deficiency. Our finding is in agreement with previous study on rice where the transcript level of both *OsLsi1* and *OsLsi2* was decreased by cadmium and copper stress [50]. Moreover, these transporters were both shown to respond to N availability where the higher and lower N inputs decreased and increased their expression respectively by supplemented Si [51]. In line with the down-regulation of Si transporters *OsLsi1* and *OsLsi2*, Mitani-Ueno et al. also showed a decline in the expression of both genes after 3 days of Si application, which was due to the accumulation of Si in the shoots [49]. As supported with the previous finding, we believe that the expression of both Si transporters (*OsLsi1* and *OsLsi2*) declined in response to S deficiency. It is worthy to notice that in our tested conditions, the expression level of *OsLsi6* responsible for loading Si from xylem to shoots sharply decreased under short-term Si application compared to the long-term Si supply. The decreases in the expression pattern of *OsLsi6* could be due to the insufficient availability of Si at the early stage of plant growth. While compared to short-term S stress, the expression of *OsLsi6* was not suppressed when S deficiency further persisted for a longer period in order to load Si to the shoot organs and therefore to cope with the lack of S.

Altogether, these data demonstrate that Si basically prepared the rice plants to tolerate short as well as long-term S deficiencies firstly by changing the expression of S transporters and secondly by modulating the expression of Si transporters, which increased the accumulation of Si to shoots to indirectly compensate the function of S.

### 3.2. Si Regulates Source to Sink Metabolic Homeostasis and Maintains the Growth of Rice under S Deficiency

During the last decade, the effect of Si on the mitigation of individual mineral deficiencies and toxicities has been extensively reported [24,27,52]. The majority of research studies dealing with cross-talk between Si and mineral nutrition showed a higher uptake or accumulation of macronutrients, whereas Si was shown to decrease the accumulation of heavy metals in the above-ground part of crop plants. However, depending on plant species, a controversial effect of Si has also been reported. As an example, Hu et al. showed a decrease in shoot P when rice plants were treated with Si, while in wheat, Si increased the shoot P concentration with a sufficient amount of P fertilizer [35]. In nitrogen-deprived rapeseed plants, Haddad et al. showed that Si increased the uptake of NO_3_ in hydroponically grown rapeseed plants as non-Si-accumulator plants [24]. In our conditions, we found that the shoot concentration of most elements was decreased in the presence of Si under both ample and S-deficient conditions except for P nutrition. These results are partially in agreement with those of Greger et al., where Si induced a decrease in the concentration of most macronutrients in both shoots and roots in different crop species such as wheat, maize, and lettuce, which was thought to be due to the dilution effect [53]. In the present study, most elements were decreased in shoots by Si supplement not only under the lack of S but also in control plants. This could be due to the dilution effect, considering that the growth of plants was enhanced by Si as supported by total mineral levels, which remained unchanged in the shoots (Supplementary Table S2).

The effect of Si was also studied on the primary metabolism of rice plants under ample S and S deprivation. Under S deficiency alone, we could detect a higher accumulation of amino acids in shoots, even though their concentration was also increased in roots. Conversely, in roots and except for the polyamines, which remained unchanged, the levels of almost all amino acids and organic acids increased under S deficiency when Si was supplemented. Moreover, in shoots, the levels of all amino acids together with isocitrate were decreased by Si supply. In *Arabidopsis*, a similar increase in putrescine and several amino acids was reported under S deficiency to keep a steady level of metabolites under N/S imbalance [54,55]. In the present work, the higher accumulation of metabolites by Si in roots and their reduction in shoots could be due to several reasons. Generally, S deficiency leads to an accumulation of amino acids [56] or NO_3_^−^ in shoots [57,58]. Looking to the total amino acid levels in the shoots indicated no difference between Si-treated and Si non-treated plants, which was also comparable with the control condition, even though their concentrations were lower in Si-treated plants. This decrease could be due to the higher growth of Si-supplied plants under the lack of S, which led to a dilution effect, meaning that the shoots were less stressed and coped better under S deficiency when supplied with Si (Supplementary Figure S2B). The lower accumulation of amino acids in shoots could also be due to dilution effect as a higher accumulation of Si maintained the growth of plants under the lack of S. In addition, amino acids seem to have contributed less to shoot osmotic potential under such circumstances [59]. Instead, amino acids or metabolites were allocated more into the roots due to the high demand of roots in response to S stress and in order to better tolerate the lack of S. We have shown a similar trend of decrease in the level of amino acids in shoots when barley plants were subjected to concomitant S deficiency and osmotic stress [23]. In this context, the role of amino acids GABA and proline in increasing plant tolerance to various abiotic stresses particularly against nutritional stresses such as S deprivation has been widely studied [60,61]. Indeed, it was shown that the exogenous application of both GABA and proline alleviated oxidative stress in rice plants subjected to salt stress [62,63]. In addition, we have recently shown that in a drought-sensitive tomato line exposed to drought stress, the levels of both GABA and proline were increased, leading to an increase in the level of polyamines, thus contributing to stress tolerance [64]. Similarly, Si was shown to mitigate the lack of Mg in maize plant by increasing the GABA concentration [27]. Interestingly, in the present study, Si increased the N content, particularly the NH_4_^+^ concentration in the roots, which could be another reason for the accumulation of other amino acids such as glutamine and proline under such circumstances. Overall, our results revealed that roots suffered from S deficiency more than shoots. While the accumulation of Si in shoot compensated for the lack of S, Si allocated the primary metabolites for roots to combat S deficiency.

Phytohormones play an important role in plant response to biotic and abiotic stresses [65,66,67]. The regulation of hormones in response to mineral nutrition deficiency has also been well investigated [38,68,69]. Plant hormones are also involved in the regulation of sulfate uptake and assimilation. The role of both cytokinins and auxins in the regulation of S metabolism was shown in *Arabidopsis* [70,71,72]. Moreover, it was shown that JA is also involved in the regulation of sulfate assimilation without affecting the expression of the sulfur-responsive promotor element [71,73]. The cross-link between ABA and S nutrition has also been shown under drought conditions due to cysteine availability [74]. Notably, in a study in tobacco plants infected by mosaic virus, the application of SA also resulted in an increase in the level of glutathione synthase. Few studies also provided evidence that Si displayed changes in the level of phytohormones such as ABA [23], and cytokinins [75]. In our research, the levels of ABA, JA, and SA increased under S deficiency, while the application of Si decreased their level. These hormones are involved in plant response to mineral deficiencies and regulate the senescence process in plants [65,76,77,78]. The lower level of phytohormones induced by Si nutrition in this study can be associated with the expression of S transporters. A decrease in the level of phytohormones ABA, JA, and SA by Si supply can decrease the level of S stress as supported by the lower expression level of *OsSULTR1;2* and *OsSULTR2;2* after 15 days of S deficiency. We have also shown in our previous works that Si decreased the level of ABA in barley plants exposed to combined S or K deprivations and osmotic stress [23,75]. In addition, we have shown that Si increased the level of JA-Ile in maize plants subjected to magnesium deficiency [27]. Altogether, these results indicate that Si lowered the level of stress by decreasing the concentrations of phytohormones, thus retarding plant senescence and maintaining plant growth and development [23].

## 4. Materials and Methods

### 4.1. Plant Material and Growth Conditions

The hydroponics experiments were conducted in a greenhouse equipped with high-pressure sodium lights (60% humidity, 16 h days at 26 °C, and 8 h night at 21 °C). Rice seeds (cv Arelate) were germinated for 12 days on vermiculite with the addition of 0.5X Hoagland solution (0.5 mM Ca(NO_3_)_2_, 0.5 mM K_2_SO_4_, 0.25 mM MgSO_4_, 0.25 mM NH_4_H_2_PO_4_, 0.25 mM CaCl_2_,2 H_2_0, 5 µM H_3_BO_3_, 5 µM MnSO_4_, 0.5 µM ZnSO_4_, 0.25 µM (NH_4_)_6_Mo_7_O_24_, 50 µM EDTA,2NaFe, 0.5 µM CuNO_3_) and were then transplanted to 7 L trays with full Hoagland, each containing 12 plants. Hydroponics solution was buffered to pH 5.9 and renewed twice a week with continuous aeration. S deficiency and Si application (2 mM) were started 20 days after transplanting in S-sufficient condition. Under S deprivation, K_2_SO_4_, MgSO_4_, MnSO_4_, and ZnSO_4_ were respectively replaced by the same concentration of KCl, MgCl_2_, MnCl_2_, and ZnCl_2_. Monosilicic acid [Si(OH)_4_] was prepared by passing sodium silicate solution through a column filled with cation-exchange resins (Amberlite, Sigma Aldrich, St. Louis, MO, USA city, state abbreviation if USA or Canada, country) according to Yin et al. [79]. Si was provided for plants at the beginning of S deficiency and each time when the solution was renewed. Samples were harvested 6 h, 12 h, 24 h, 3 days, and 15 days after the application of Si and S deprivation (each time, four biological replicates containing three pooled plants). For the last harvest, the total fresh weight of the shoots and the roots were determined.

### 4.2. RNA Extraction and Gene Expression Analysis

The root and leaf samples (100 mg and 70 mg, respectively) of rice plants harvested for each time point (6 h, 12 h, 24 h, 3 days and 15 days) were ground to a fine powder in the presence of liquid nitrogen, and the total RNA was extracted using a Nucleospin^®^8 RNA kit following the manufacturer’s protocol (Macherey-Nagel, Düren, Germany). The quality and yield of all RNA samples were analyzed and checked in a 4200 Tapestation (Agilent Technologies, Santa Clara, CA, USA), followed by DNase treatment and cDNA synthesis from 1 µg RNA using an iScript gDNA clear cDNA synthesis kit (Bio-Rad, Hercules, CA, USA). Quantitative RT-PCR (qPCR) analysis was performed in a total volume of 10 µL using Universal SYBR Green Supermix (Bio-Rad, Hercules, CA, USA) in a Real-Time PCR Detection System (Bio-Rad, Hercules, CA, USA). The qPCR reactions were performed in technical triplicates using independent cDNA reactions for each biological replicate and 300 nM of gene-specific primer pairs. Specific primers for all candidate genes were designed using Primer3 software (version 0.4.0) and are listed in Supplementary Table S3. The thermal cycler protocol was 98 °C for 3 min, 40 cycles of 98 °C for 15 s, 60 °C for 30 s, 72 °C for 15 s, and a final 5-min extension at 72 °C. The expression of all candidate genes was normalized against four rice reference genes, namely *OsActin*, *OsEF1α*, *OsTubulin,* and *OsGAPDH*. All qPCR expression data were acquired and analyzed using CFX Maestro Software Version 1.0 (Bio-Rad, Hercules, CA, USA). The relative gene expression at each time point was normalized to the expression of plants that had received ample S and no Si (+S-Si). Statistics were performed for each time point independently.

### 4.3. Determination of Mineral Elements

Elemental analysis was performed according to Maillard et al. (2016) in the PLATIN’ (Plateau d’Isotopie de Normandie) core facility [80]. Elements were quantified by high-resolution inductively coupled plasma mass spectrometry (HR ICP-MS, Thermo Scientific, Waltham, MA, USA, Element 2TM) with prior microwave acid sample digestion (Multiwave ECO, Anton Paar, les Ulis, France) (800 µL of concentrated HNO_3_, 200 µL of H_2_O_2_, and 1 mL of Milli-Q water for 40 mg DW). For the determination by high-resolution inductively coupled plasma mass spectrometry (HR ICP-MS), all the samples were spiked with two internal standard solutions of gallium and rhodium for final concentrations of 10 µg L^−1^ and 2 µg L^−1^, respectively, diluted to 50 mL with Milli-Q water to obtain solutions containing 2.0% (*v*/*v*) of nitric acid; then, they were filtered at 0.45 µm using a Teflon filtration system (Filtermate, Courtage Analyses Services, Mont-Saint-Aignan, France). The quantification of each element was performed using external standard calibration curves.

For Si determination, 8 mL of 0.1 M Tiron solution buffered at pH 10.5 was added to 25 mg of dry material, which was continuously shaken for at least 4 h at 65 °C in a shaker incubator (Infors HT, Minitron, Bottmingen, Switzerland). After cooling, 7 mL of H_2_O_2_ (Roth, Lagny-sur-Marne, France) was added to destroy Tiron. The tubes were shaken horizontally in a water bath at 85 °C until the solution turned colorless. The samples were then centrifuged at 4000 rpm at 25 °C for 10 min before analysis. The elements were analyzed by Inductively Coupled Plasma Optical Emission Spectrometry (iCAP 6500 dual OES spectrometer, Thermo Scientific, Waltham, MA, USA) by using Yttrium solution (1 ppm, Merck, Darmstadt, Germany) as an internal standard.

Analysis of N was performed using an elemental FLASH 2000 CHNS analyzer (Thermo Scientific, Waltham, MA, USA) according to the manufacturer’s instructions from 2.5 mg of homogenized and lyophilized plant material.

Ammonium, nitrate, and sulfate extraction was adapted from Abdallah et al. (2010) [81]. First, 20 mg of dry material was extracted with 1 mL of ethanol/water 50:50 (*v*/*v*), sonicated for 15 min, and incubated for 1 h at 40 °C. After centrifugation (14,000 rpm for 15 min at 4 °C), the supernatant was transferred to a new Eppendorf tube, and 1 mL of ddH_2_O was added to the pellet. After an incubation at 95 °C for 1 h, the second supernatant was added to the first one and was evaporated and solubilized in 1 mL of ddH_2_O. The anions and cations were determined using high-performance liquid chromatography with a conductivity detector (Dionex ICS5000+, Thermo Scientific, Villebon-sur-Yvette, France). Gradients of potassium hydroxide (KOH) and methane sulfonic acid (MSA) were used to perform the separation between anions and cations over an analytical column AS19 and CS12, respectively.

### 4.4. Determination of Primary Metabolites

For amino acid determination, 10 mg of lyophilized dry matter was extracted with a solution containing 400 µL of MeOH and 0.250 nmol/µL Norvaline, which was used as the internal standard (Sigma Aldrich, St. Louis, MO, USA). The extract was stirred for 15 min, and it was then re-suspended with 200 µL of chloroform (agitation for 5 min) and 400 µL of double-distilled water (ddH_2_O). After centrifugation (12,000 rpm, 10 °C, 5 min), the supernatant was recovered, evaporated, and dissolved in 100 µL of ddH_2_O. Derivatization was performed using an Ultra Derivatization Kit AccQ tag (Waters Corp, Milford, MA, USA), following the manufacturer’s protocol (Waters Corp, Milford, MA, USA). The amino acid profile was determined by ultra-performance liquid chromatography coupled with a photodiode array detector (UPLC/PDA) H-Class system with an ethylene bridge hybrid (BEH) C18 100 × 2.1 mm column (pore size: 1.7 µm). Since methionine could not be determined using this method, it was extracted and analyzed according to Ali et al. 2018 [64].

Organic acid extraction was conducted using 30 mg of frozen ground fresh leaves and roots, which were weighed in 2 mL Eppendorf tubes; then, 500 µL of cold water/methanol 70:30 *v*/*v* (−20 °C) containing 0.1% of perchloric acid (*v*/*v*) solvent and 50 ng of isotopically labeled succinic acid (CLM-1084-PK, Cambridge Isotope Laboratories, Tewksbury, MA, USA) were added. Samples were shaken with vortex for 20 min. Then, they were centrifuged using an Eppendorf Centrifuge 5427 R (Eppendorf, Hamburg, Germany) for 20 min at 12,700 rpm at 4 °C. Supernatants were collected and introduced in a new 2 mL Eppendorf tube. A second extraction was performed by adding 500 µL of ddH_2_O + 0.1% perchloric acid (*v*/*v*) + 50 ng of isotopically labelled succinic acid to the pellet, after which it was shaken for 5 min with vortex and centrifuged for 20 min at 12,700 rpm at 4 °C. Supernatants were mixed and centrifuged for 10 min in order to eliminate suspended particles. Finally, supernatants were diluted twice with ddH_2_O + 0.1% formic acid (*v*/*v*) and introduced in 2 mL LC-MS vials. The separation and detection were achieved using a Nexera X2 UHPLC system (Shimadzu, Kyoto, Japan) coupled to a QTrap 6500+mass spectrometer TM (Sciex, Concord, ON, Canada) equipped with an IonDrive turbo V electrospray (ESI) source. A Phenomenex Luna^®^Omega PS C18 (100 × 2.1 mm, 1.6 µm) column (Torrance, CA, USA) was used to profile the organic acids. The mobile phase, comprising water containing 0.5% formic acid (A) and methanol:water (70:30 *v*/*v*) containing 0.5% formic acid (B) was applied with the optimized gradient elution as follows: 100% A at 0–1 min, 100–20% A at 1–4 min, 20–0% A at 4–6.5 min, 0% A at 6.5–7.5 min, 0–100% A at 7.5–7.9 min, and 100% A at 7.9–10 min. The flow rate was kept at 0.3 mL/min, and the column temperature was maintained at 40 °C. The injection volume for both columns was 10 µL, and samples were maintained at 10 °C. The ESI source was used in negative ionization, the source voltage was set to 2.5 kV, and the cone voltage was 30 V, whilst the source temperature was maintained at 130 °C with a cone gas flow of 20 L/h. The desolvation temperature was at 500 °C with a desolvation gas flow of 900 L/h. Leucine–enkephalin was used as lockmass reference, (ion at m/z 556.2771 in positive mode), which was introduced by a lockspray at 10 µL min^−1^ for real-time data calibration. The MSE data were acquired in centroid mode using a scan range 50–800 Da and a scan time of 0.1 s; the resolution was set at 20,000 full width half maximum (FWHM), and a collision energy ramp at 40–80 V.

Soluble sugar determination was undertaken according to the method described by Kim et al. [82]. Ten mg lyophilized shoot material was homogenized in liquid nitrogen, dissolved in 0.75 mL of 80% (*v*/*v*) ethanol, and incubated at 80 °C for 30 min. Crude extracts were decanted for 15 min at retention time (RT), centrifuged at 14,000 rpm for 10 min at 4 °C, and concentrated in a Speed Vac concentrator (Thermo Scientific, Waltham, MA, USA) at 45 °C for 180 min. The pellet was re-suspended in 0.75 mL of deionized water and incubated at 80 °C for 30 min. After centrifugation, the second supernatant was added to the first, concentrated, and resuspended in 0.5 mL of ddH_2_O. Hexokinase (HK), phosphoglucoisomerase (PGI), and beta-fructosidase were added successively to measure glucose (Glc), fructose (Fru), and sucrose (Suc), which were determined by spectrophotometry at 340 nm (SpectraMax i3x, Molecular Devices, San Jose, CA, USA).

Polyamine extraction was achieved using 20 mg of frozen ground leaves that were weighed in a 2 mL Eppendorf tube (Eppendorf, Hamburg, Germany). Extraction was carried out by adding 1 mL of a solution of 70% H_2_O/29% MeOH/1.0% formic acid (*v*/*v*/*v*) at −20 °C. Next, the tubes were stirred at room temperature for 30 min and then centrifuged at 4 °C (16,000 rpm), and the supernatant was transferred into a new Eppendorf tube. The supernatant was transferred to a LC/MS vial for analysis. Polyamines were analyzed by a UHPLC–MS/MS system. The separation and detection were achieved using a Nexera X2 UHPLC system (Shimadzu, Kyoto, Japan) coupled to a QTrap 6500+ mass spectrometer (Sciex, Concord, ON, Canada) equipped with an IonDriveTM turbo V electrospray (ESI) source.

### 4.5. Determination of Phytohormones

Salicylic acid (SA), jasmonic acid (JA), jasmonoyl-isoleucine (JA-Ile), and abscisic acid (ABA) were all purchased from OlchemIn (Olomouc, Czech Republic). Acidic phytohormones, ABA, JA, JA-Ile and SA were analyzed by a UHPLC-MS/MS system. Then, 10 mg of FW samples were extracted with 70% methanol, 29% H2O, 1% formic acid containing isotope-labeled internal standards, and centrifuged at 12,600 rpm to collect the supernatant. After evaporation (SPE Dry 96, Biotage, Uppsala, Sweden), the extract was re-suspended in 2% formic acid solution and purified using a SPE ABN (Solid Phase Extraction, Acidic, Basic and Neutral Analytes) express plate of 30 mg/mL (Biotage, Uppsala, Sweden). The phytohormones were eluted with methanol, and samples were evaporated and re-suspended in 200 µL of 0.1% formic acid solution before injection into the system. The separation and detection were achieved using a Nexera X2 UHPLC system (Shimadzu, Kyoto, Japan) coupled to a QTrap 6500+mass spectrometer TM (Sciex, Concord, ON, Canada) equipped with an IonDrive turbo V electrospray (ESI) source. Phytohormone separation was carried out by injecting a 2 µL sample into a Kinetex Evo C18 core–shell column (100 × 2.1 mm, 2.6 µm, Phenomenex, Torrance, CA, USA) at a flow rate of 0.7 mL/min, and the column temperature was maintained at 40 °C. The mobile phases were composed of solvent A Milli-Q water (18 Ω, Millipore, Burlington, MA, USA) containing 0.1% formic acid (LCMS grade, Fluka analytics, Munich, Germany), and solvent B acetonitrile LCMS grade (Fisher Optima, Loughborough, UK) containing 0.1% formic acid. The gradient elution started with 1% B, 0.0–5.0 min 60% B, 5.0–5.5 min 100% B, 5.5–7.0 min 100% B, 7.0–7.5 min 1% B, and 7.5–9.5 min 1% B. The analysis was performed in scheduled MRM mode in positive mode.

### 4.6. Statistical Analysis

Data are represented as the mean ± standard deviation (SD) or standard error of the mean (SEM) for *n* = 4. The analysis of variance (ANOVA) and the post-hoc Student–Newman–Keuls (SNK) (R software) were employed to analyze the data and marked by different letters when significantly different (*p* < 0.05). Two-way ANOVA was also performed on biomass parameters and results of elemental, metabolic and hormonal analyses to detect the interaction between Si and S treatments (Supplemental Table S1).

## 5. Conclusions

In the present work, we demonstrated a distinct metabolic regulation in rice plants treated with Si under long-term S deficiencies. The expression of Si transporters *OsLsi1* and *OsLsi2* reduced when S stress was further prolonged, while that of *OsLsi6* was not suppressed in response to long-term S deficiency in order to further load the Si from xylem to shoot organs. Even though the S transporters were suppressed by Si supply under long-term S stress, a higher accumulation of Si in the shoots compensated for the lack of shoot S by balancing the source-sink metabolite homeostasis. In this context, Si effectively decreased the level of stress as supported by the lower accumulation of stress phytohormones and therefore maintaining plant growth and development under S deprivation.

## Figures and Tables

**Table 1:** Influence of silicon application on the concentrations of macroelements in rice roots and shoots exposed to sulfur deficiency. Plants were grown in hydroponic culture under either low (0 mM) or normal S (1.5 mM) as well as 2 mM of Si supply. Roots and shoots were harvested for elemental analysis after 15 days of treatments. Concentrations are expressed as mean ± SD in mg g^−1^ of dry weight (DW). Different letters denote significant difference according to ANOVA followed by SNK test (*p* < 0.05; *n* = 4). ^ns^ denotes non-significant changes. Abbreviations are S: sulfur; Si: silicon; N: nitrogen; P: phosphorous; K: potassium; Mg; magnesium; Ca: calcium; No_3_^−^: nitrate; NH_4_^+^: ammonium and SO_4_^2^^−^: sulfate.

		Roots				Shoots			
		+S-Si	+S+Si	-S-Si	-S+Si	+S-Si	+S+Si	-S-Si	-S+Si
**Macro-Elements**	**S**	3.79 ± 0.18 ^a^	3.90 ± 0.19 ^a^	2.68 ± 0.08 ^b^	2.59 ± 0.15 ^b^	4.37 ± 0.39 ^a^	3.29 ± 0.24 ^b^	3.08 ± 0.25 ^b^	2.11 ± 0.12 ^c^
	**Si**	3.00 ± 0.00 ^b^	11.50 ± 1.00 ^a^	2.50 ± 0.58 ^b^	10.50 ± 1.00 ^a^	3.00 ± 0.82 ^c^	84.50 ± 3.32 ^a^	2.75 ± 0.50 ^c^	69.75 ± 5.97 ^b^
	**N**	28.83 ± 1.76 ^ns^	27.94 ± 0.98 ^ns^	28.61 ± 0.93 ^ns^	27.75 ± 0.44 ^ns^	36.99 ± 0.65 ^a^	31.43 ± 0.39 ^b^	36.03 ± 1.57 ^a^	28.54 ± 1.61 ^c^
	**P**	9.00 ± 0.17 ^a^	7.64 ± 0.38 ^b^	8.01 ± 0.35 ^b^	6.98 ± 0.35 ^c^	10.26 ± 0.53 ^a^	6.42 ± 0.37 ^c^	8.68 ± 0.96 ^b^	5.43 ± 0.15 ^d^
	**K**	29.42 ± 1.60 ^a^	28.00 ± 0.99 ^ab^	25.51 ± 2.01 ^b^	27.76 ± 0.35 ^ab^	37.67 ± 2.93 ^ns^	35.14 ± 1.93 ^ns^	34.12 ± 2.19 ^ns^	34.77 ± 3.62 ^ns^
	**Mg**	2.27 ± 0.20 ^b^	3.10 ± 0.77 ^a^	2.27 ± 0.13 ^b^	1.91 ± 0.18 ^b^	4.76 ± 0.26 ^a^	2.51 ± 0.16 ^b^	4.58 ± 0.52 ^a^	2.25 ± 0.19 ^b^
	**Ca**	1.51 ± 0.20 ^ns^	1.53 ± 0.19 ^ns^	1.51 ± 0.09 ^ns^	1.33 ± 0.09 ^ns^	5.09 ± 0.55 ^a^	2.88 ± 0.28 ^b^	5.36 ± 0.83 ^a^	2.73 ± 0.35 ^b^
**Ions**	**NO_3_^−^**	12.84 ± 3.07 ^a^	15.01 ± 0.72 ^a^	8.74 ± 0.32 ^b^	8.20 ± 0.67 ^b^	5.18 ± 0.97 ^ab^	6.71 ± 0.61 ^a^	3.18 ± 2.12 ^bc^	2.04 ± 1.85 ^c^
	**NH_4_^+^**	0.19 ± 0.02 ^ab^	0.18 ± 0.01 ^b^	0.20 ± 0.02 ^ab^	0.22 ± 0.01^a^	0.19 ± 0.02 ^a^	0.12 ± 0.01 ^b^	0.21 ± 0.02 ^a^	0.11 ± 0.02 ^b^
	**SO_4_^2−^**	3.02 ± 0.38 ^b^	3.49 ± 0.25 ^a^	0.92 ± 0.17 ^c^	1.10 ± 0.24 ^c^	4.13 ± 0.92 ^a^	2.82 ± 0.49 ^b^	1.63 ± 0.24 ^c^	0.71 ± 0.13 ^d^

## Supplementary Materials

Supplementary materials can be found at https://www.mdpi.com/1422-0067/21/10/3677/s1. Supplementary Figure S1: Effect of Si on primary metabolites under control condition; Supplementary Figure S2: Total amino acid accumulation in roots and shoots; Supplementary Table S1: Two-way ANOVA; Supplementary Table S2: Total accumulation of elements in roots and shoots; Supplementary Table S3: List of primers.

## Abbreviations

ABA	Abscisic acid
GABA	Gamma-aminobutyric acid
JA	Jasmonic acid
JA-Ile	Jasmonoyl-isoleucine
SA	Salicylic acid
