# Supplementary material for: Silicon Regulates Source to Sink Metabolic Homeostasis and Promotes Growth of Rice Plants under Sulfur Deficiency
Source: Int J Mol Sci. 2020 May 23;21(10):3677. doi: 10.3390/ijms21103677 (PMC7279143; doi:10.3390/ijms21103677)
Supplement: Supplementary file 1 [file ijms-21-03677-s001.pdf]

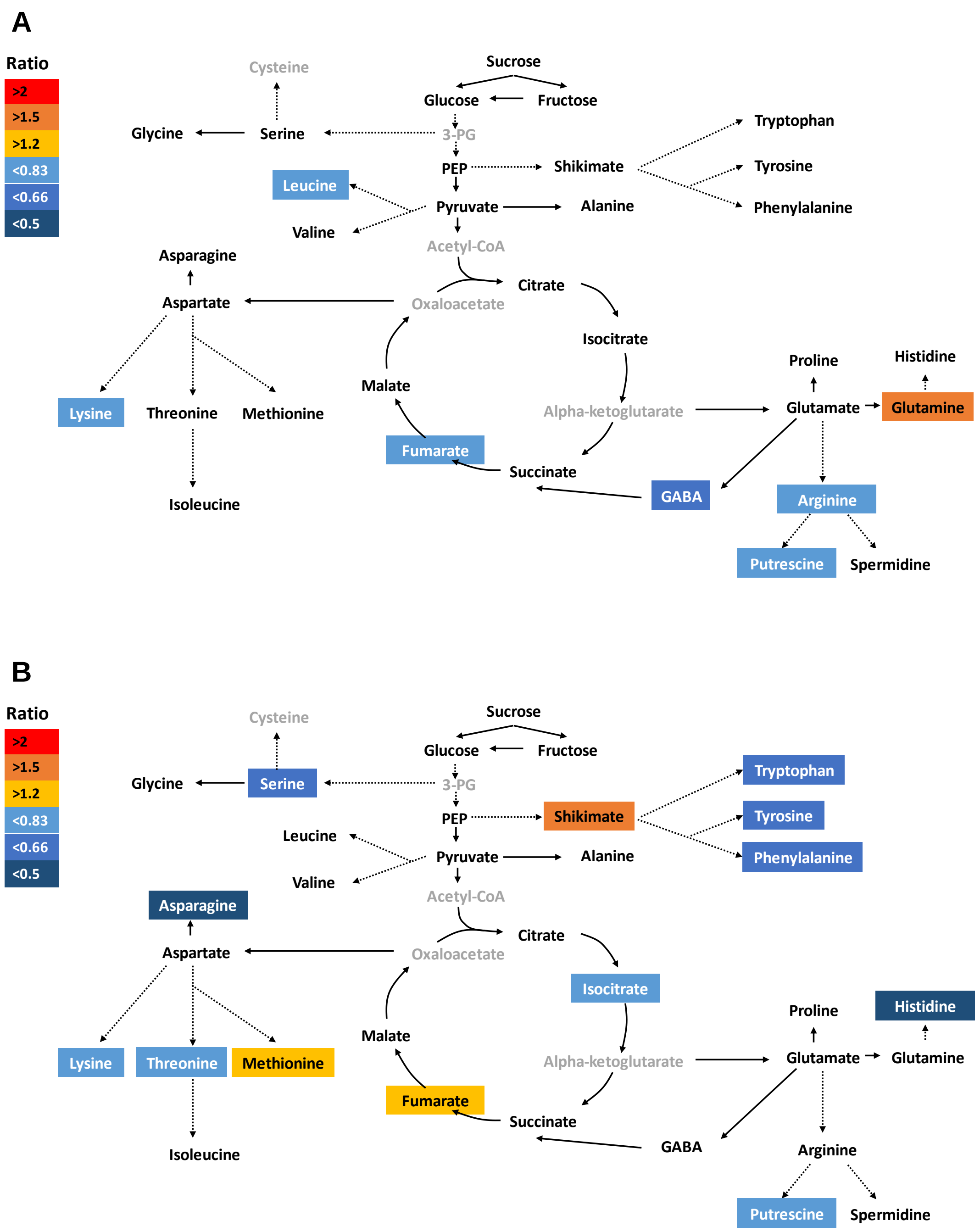


**Supplementary Figure S1. Influence of silicon application on the concentrations of primary metabolites of rice roots and shoots under ample S condition.** Schematic representation of primary metabolites under ample S and in response to Si application. (A) root and (B) shoot. Plants were grown in hydroponic culture under normal S (1.5 mM) as well as 2 mM of Si. Roots and shoots were harvested for metabolite profiling after 15 days of treatments. Metabolites were coloured according to the ratio of normal S supply treated with Si to normal S supply without Si treatment (light blue to dark blue: decrease; yellow to red: increase). The grey colour indicated the unquantified metabolites. Only the metabolites which presented statistically significant differences were presented. Solid arrows denote single reactions, whereas dotted arrows indicate that some intermediate metabolites are not shown. Statistical analysis was done according to ANOVA followed by SNK test ($p<0.05$; $n=4$).

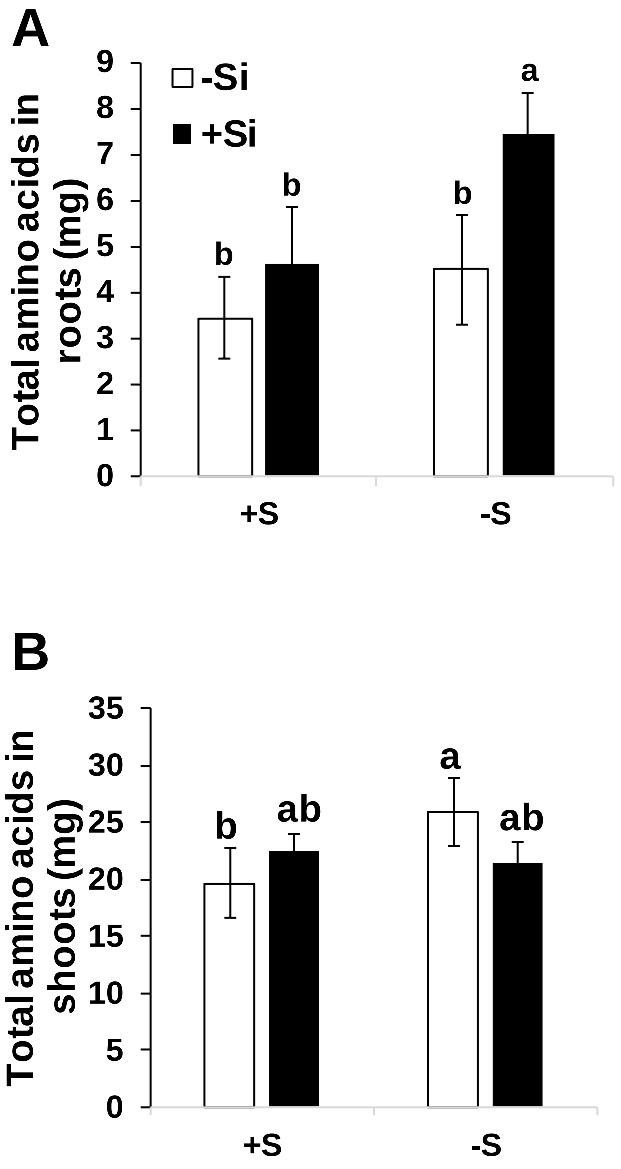


**Supplementary Figure S2: Influence of silicon application on the total amino acid accumulation in rice shoots and roots exposed to S deficiency.** (A) Total amino acid in roots and (B) total amino acid in shoots. Plants were grown in hydroponic culture under either low (0 mM) or normal S (1.5 mM) as well as 2 mM of Si. Roots and shoots were harvested after 15 days of treatment for amino acid analysis. Data correspond to the accumulation of total amino acids in three pooled plants in mg. Different letters denote significant difference according to ANOVA followed by SNK test ($p<0.05$; n=4).

**Supplementary Table S1:** Results of Two-Way ANOVA examining the effects of Sulfur, Silicon and their interaction on roots and shoot biomass as well as macro-elements, metabolites and phytohormones in plants exposed to short and long-term S deficiency and Si application.

| Shoot | Fresh weight (g FW) | | K (mg $g^{-1}$ DW) | | S (mg $g^{-1}$ DW) | | Si (mg $g^{-1}$ DW) | | P (mg $g^{-1}$ DW) | |
|---|---|---|---|---|---|---|---|---|---|---|
| | *F-value* | *Pr(>F)* | *F-value* | *Pr(>F)* | *F-value* | *Pr(>F)* | *F-value* | *Pr(>F)* | *F-value* | *Pr(>F)* |
| **Sulfur** | 0.063 | 0.802 | 2.032 | 0.18 | 84.426 | 8.87e-07 *** | 18.95 | 0.00094 *** | 19.41 | 0.000857 *** |
| **Silicon** | 29.622 | 2.21e-06 *** | 0.466 | 0.508 | 58.007 | 6.20e-06 *** | 1857.03 | 1.58e-14 *** | 148.48 | 4.08e-08 *** |
| **Sulfur:Silicon** | 0.011 | 0.919 | 1.332 | 0.271 | 0.175 | 0.683 | 17.7 | 0.00122 ** | 0.987 | 0.340052 |

| Root | Fresh weight (g FW) | | K (mg $g^{-1}$ DW) | | S (mg $g^{-1}$ DW) | | Si (mg $g^{-1}$ DW) | | P (mg $g^{-1}$ DW) | |
|---|---|---|---|---|---|---|---|---|---|---|
| | *F-value* | *Pr(>F)* | *F-value* | *Pr(>F)* | *F-value* | *Pr(>F)* | *F-value* | *Pr(>F)* | *F-value* | *Pr(>F)* |
| **Sulfur** | 7.517 | 0.008804 *** | 9.028 | 0.0110 * | 237.026 | 2.88e-09 *** | 10.805 | 0.00724 ** | 25.621 | 0.000279 *** |
| **Silicon** | 13.581 | 0.000623 *** | 0.362 | 0.5588 | 0.011 | 0.917 | 395.413 | 5.69e-10 *** | 53.441 | 9.34e-06 *** |
| **Sulfur:Silicon** | 0.339 | 0.563614 | 7 | 0.0213 * | 1.517 | 0.242 | 0.363 | 0.55925 | 1.006 | 0.335692 |

| Ca (mg $g^{-1}$ DW) | | Mg (mg $g^{-1}$ DW) | | N (mg $g^{-1}$ DW) | | $NH_4^+$ (mg $g^{-1}$ DW) | | $NO_3^-$ (mg $g^{-1}$ DW) | | $SO_4^{2-}$ (mg $g^{-1}$ DW) | |
|---|---|---|---|---|---|---|---|---|---|---|---|
| *F-value* | *Pr(>F)* | *F-value* | *Pr(>F)* | *F-value* | *Pr(>F)* | *F-value* | *Pr(>F)* | *F-value* | *Pr(>F)* | *F-value* | *Pr(>F)* |
| 0.05 | 0.826 | 1.94 | 0.189 | 10.527 | 0.00703 ** | 0.684 | 0.425 | 23.986 | 0.000625 *** | 73.41 | 1.85e-06 *** |
| 79.159 | 1.25e-06 *** | 212.569 | 5.38e-09 *** | 121.247 | 1.25e-07 *** | 87.797 | 7.2e-07 *** | 0.2 | 0.66562 | 17.16 | 0.00136 ** |
| 0.628 | 0.444 | 0.073 | 0.792 | 2.656 | 0.12913 | 1.899 | 0.193 | 9.156 | 0.012767 * | 0.53 | 0.4804 |

| Ca (mg $g^{-1}$ DW) | | Mg (mg $g^{-1}$ DW) | | N (mg $g^{-1}$ DW) | | $NH_4^+$ (mg $g^{-1}$ DW) | | $NO_3^-$ (mg $g^{-1}$ DW) | | $SO_4^{2-}$ (mg $g^{-1}$ DW) | |
|---|---|---|---|---|---|---|---|---|---|---|---|
| *F-value* | *Pr(>F)* | *F-value* | *Pr(>F)* | *F-value* | *Pr(>F)* | *F-value* | *Pr(>F)* | *F-value* | *Pr(>F)* | *F-value* | *Pr(>F)* |
| 1.755 | 0.21 | 8.367 | 0.0135 * | 0.131 | 0.723 | 8.417 | 0.0133 * | 45.351 | 2.09e-05 *** | 272.056 | 1.31e-09 *** |
| 1.133 | 0.308 | 1.307 | 0.2752 | 2.422 | 0.146 | 0.029 | 0.8673 | 1.011 | 0.334 | 5.651 | 0.0349 * |
| 1.634 | 0.225 | 8.455 | 0.0131 * | 0.001 | 0.979 | 3.524 | 0.085 | 2.796 | 0.12 | 1.114 | 0.312 |

| Total amino acids (mg) | | Glutamine (mg $g^{-1}$ DW) | | Proline (mg $g^{-1}$ DW) | | GABA (mg $g^{-1}$ DW) | | Citrate (mg $g^{-1}$ FW) | | Isocitrate (mg $g^{-1}$ FW) | |
|---|---|---|---|---|---|---|---|---|---|---|---|
| *F-value* | *Pr(>F)* | *F-value* | *Pr(>F)* | *F-value* | *Pr(>F)* | *F-value* | *Pr(>F)* | *F-value* | *Pr(>F)* | *F-value* | *Pr(>F)* |
| 5.027 | 0.0465 * | 0.067 | 0.8005 | 18.776 | 0.001188 ** | 14.24 | 0.003077 ** | 7.845 | 0.016 * | 4.409 | 0.05757 |
| 0.157 | 0.6995 | 12.915 | 0.00422 ** | 23.401 | 0.000521 *** | 33.74 | 0.000118 *** | 0.186 | 0.674 | 43.69 | 2.5e-05 *** |
| 7.91 | 0.0169 * | 6.272 | 0.02927 * | 1.725 | 0.215812 | 11.33 | 0.006303 ** | 0.004 | 0.948 | 11.243 | 0.00575 ** |

| Total amino acids (mg) | | Glutamine (mg $g^{-1}$ DW) | | Proline (mg $g^{-1}$ DW) | | GABA (mg $g^{-1}$ DW) | | Citrate (mg $g^{-1}$ FW) | | Isocitrate (mg $g^{-1}$ FW) | |
|---|---|---|---|---|---|---|---|---|---|---|---|
| *F-value* | *Pr(>F)* | *F-value* | *Pr(>F)* | *F-value* | *Pr(>F)* | *F-value* | *Pr(>F)* | *F-value* | *Pr(>F)* | *F-value* | *Pr(>F)* |
| 13.437 | 0.00372 ** | 5.625 | 0.037 * | 9.681 | 0.00990 ** | 2.681 | 0.129838 | 63.88 | 3.8e-06 *** | 17.89 | 0.001169 ** |
| 14.884 | 0.00266 ** | 41.783 | 4.65e-05 *** | 19.243 | 0.00109 ** | 0.196 | 0.666673 | 13.27 | 0.00337 ** | 16.45 | 0.001594 ** |
| 2.535 | 0.13965 | 0.046 | 0.834 | 10.428 | 0.00803 ** | 24.797 | 0.000416 *** | 11.23 | 0.00577 ** | 21.37 | 0.000587 *** |

| Abscisic acid (µg $g^{-1}$ FW) | | Salicylic acid (µg $g^{-1}$ FW) | | Jasmonic acid (µg $g^{-1}$ FW) | | Jasmonic acid-Isoleucine (µg $g^{-1}$ FW) | |
|---|---|---|---|---|---|---|---|
| *F-value* | *Pr(>F)* | *F-value* | *Pr(>F)* | *F-value* | *Pr(>F)* | *F-value* | *Pr(>F)* |
| 54.68 | 8.33e-06 *** | 6.107 | 0.0310 * | 1.689 | 0.223 | 51.65 | 5.16e-05 *** |
| 23.81 | 0.000379 *** | 6.596 | 0.0261 * | 0.898 | 0.366 | 48.8 | 6.42e-05 *** |
| 11.03 | 0.006094 ** | 4.198 | 0.0651 | 0.301 | 0.595 | 18.66 | 0.00194 ** |

**Supplementary Table S2: Influence of silicon application on the total level of macro-elements in roots and shoots of rice plant exposed to sulfur deficiency.** Plants were grown in hydroponic culture under either low (0 mM) or normal S (1.5 mM) as well as 2 mM of Si. Roots and shoots were harvested after 15 days of treatment for elemental analysis. Data are expressed as mean ± SD in mg for three pooled plants. Different letters denote significant difference according to ANOVA followed by SNK test ($p<0.05$; n=4). Abbreviations are: S: sulfur; Si: silicon; N: nitrogen; P: phosphorous; K: potassium; Mg; magnesium; Ca: calcium; $No_3^-$: nitrate; $NH_4^+$: ammonium and $SO_4^{2-}$: sulfate.

| | | Roots | | | | Shoots | | | |
|---|---|---|---|---|---|---|---|---|---|
| | | +S-Si | +S+Si | -S-Si | -S+Si | +S-Si | +S+Si | -S-Si | -S+Si |
| Macro-elements | S | 1.95 ± 0.39 $^{ab}$ | 2.48 ± 0.43 $^{a}$ | 1.64 ± 0.27 $^{b}$ | 2.08 ± 0.18 $^{ab}$ | 9.6 ± 1.59 $^{a}$ | 10.23 ± 0.86 $^{a}$ | 7.48 ± 0.94 $^{b}$ | 6.69 ± 0.66 $^{b}$ |
| | Si | 1.67 ± 0.25 $^{b}$ | 7.34 ± 1.64 $^{a}$ | 1.52 ± 0.38 $^{b}$ | 8.48 ± 1.34 $^{a}$ | 6.41 ± 0.93 $^{c}$ | 263.73 ± 28.26 $^{a}$ | 6.64 ± 1.15 $^{c}$ | 221.62 ± 31.13 $^{b}$ |
| | N | 14.90 ± 3.50 $^{b}$ | 17.69 ± 2.43 $^{b}$ | 17.58 ± 3.28 $^{b}$ | 22.34 ± 2.11 $^{a}$ | 81.96 ± 16.25 $^{ns}$ | 97.95 ± 8.09 $^{ns}$ | 87.53 ± 9.12 $^{ns}$ | 90.72 ± 11.80 $^{ns}$ |
| | P | 4.64 ± 1.01 $^{ns}$ | 4.84 ± 0.63 $^{ns}$ | 4.89 ± 0.65 $^{ns}$ | 5.60 ± 0.35 $^{ns}$ | 22.83 ± 5.18 $^{ns}$ | 19.98 ± 1.57 $^{ns}$ | 21.15 ± 3.40 $^{ns}$ | 17.19 ± 1.30 $^{ns}$ |
| | K | 15.24 ± 3.83 $^{b}$ | 17.71 ± 2.04 $^{b}$ | 15.66 ± 2.93 $^{b}$ | 22.32 ± 1.69 $^{a}$ | 83.87 ± 19.24 $^{ns}$ | 109.66 ± 12.44 $^{ns}$ | 82.96 ± 10.01 $^{ns}$ | 110.50 ± 16.47 $^{ns}$ |
| | Mg | 1.16 ± 0.22 $^{b}$ | 1.97 ± 0.59 $^{a}$ | 1.39 ± 0.23 $^{ab}$ | 1.53 ± 0.18 $^{ab}$ | 10.51 ± 2.00 $^{a}$ | 7.82 ± 0.83 $^{b}$ | 11.14 ± 1.72 $^{a}$ | 7.15 ± 1.06 $^{b}$ |
| | Ca | 0.77 ± 0.17 $^{ns}$ | 0.98 ± 0.23 $^{ns}$ | 0.92 ± 0.13 $^{ns}$ | 1.08 ± 0.15 $^{ns}$ | 11.25 ± 2.49 $^{ab}$ | 8.95 ± 0.74 $^{b}$ | 13.07 ± 2.57 $^{a}$ | 8.61 ± 0.87 $^{b}$ |
| Ions | $NO_3^-$ | 6.78 ± 2.46 $^{b}$ | 9.51 ± 1.34 $^{a}$ | 5.36 ± 0.99 $^{b}$ | 6.60 ± 0.85 $^{b}$ | 11.48 ± 3.06 $^{b}$ | 20.82 ± 1.28 $^{a}$ | 10.56 ± 0.27 $^{b}$ | 8.55 ± 5.26 $^{b}$ |
| | $NH_4^+$ | 0.10 ± 0.03 $^{b}$ | 0.11 ± 0.01 $^{b}$ | 0.12 ± 0.02 $^{b}$ | 0.17 ± 0.01 $^{a}$ | 0.42 ± 0.07 $^{ab}$ | 0.37 ± 0.05 $^{b}$ | 0.51 ± 0.06 $^{a}$ | 0.36 ± 0.08 $^{b}$ |
| | $SO_4^{2-}$ | 1.56 ± 0.43 $^{b}$ | 2.21 ± 0.38 $^{a}$ | 0.56 ± 0.12 $^{c}$ | 0.88 ± 0.19 $^{c}$ | 8.94 ± 1.81 $^{a}$ | 8.76 ± 1.58 $^{a}$ | 3.93 ± 0.56 $^{b}$ | 2.22 ± 0.28 $^{b}$ |

**Supplementary Table S3:** List of primers used for qRT-PCR.

| GENE | FORWARD PRIMER | REVERSE PRIMER | AMPLICON SIZE (BP) |
|---|---|---|---|
| *OsLsi1* | ACCTACACCTTCATCCGCTT | TCGTCGTCCTATCACACTTGG | 154 |
| *OsLsi2* | CCGTCCTCTCCGTCATCATCC | CTTCGCCACCTCATCACCCA | 86 |
| *OsLsi6* | ATCTACGGCGAGGACATGAAG | GGAAGAAGGCGAAGGAGAGG | 145 |
| *OsSULTR1;1* | GCCGATGTCAAAGAAGGTGT | CAGAGGCTGGCAATGGTG | 126 |
| *OsSULTR1;2* | TTCATCTGTGGAGTATGGCTTG | ATTAGCATCCCTGGCACCTT | 168 |
| *OsSULTR2;1* | TCGTCTTCACCGTCACCTTC | GAAATCCACCAGGAAACCCAAC | 80 |
| *OsSULTR2;2* | ATGCGACAAGAGCGGATAAG | TCACAGCACAAACAAGAGCA | 141 |
| *OsActin* | TCGTGAGAAGATGACCCAGA | ACCAGAGTCCAACACAATACC | 124 |
| *Osβ-tubulin* | ACCGTGCCCTTACTGTTCCT | CCTCCTTGGTGCTCATCTTTCC | 135 |
| *OsEF1α* | AAGATGATTCCCACCAAGCCC | ACAGCCACCGTTTGCCTC | 98 |
| *OsGAPDH* | CTGCCTTGCTCCACTTGCC | AGGGTCCATCAACGGTCTTCT | 110 |
